# Dietary fungi in cancer immunotherapy: From the perspective of gut microbiota

**DOI:** 10.3389/fonc.2023.1038710

**Published:** 2023-03-08

**Authors:** Yibing Wei, Dingka Song, Ran Wang, Tingting Li, Hui Wang, Xiaoguang Li

**Affiliations:** ^1^ State Key Laboratory of Oncogenes and Related Genes, Center for Single-Cell Omics, School of Public Health, Shanghai Jiao Tong University School of Medicine, Shanghai, China; ^2^ College of Medical Technology, Shanghai University of Medicine and Health Sciences, Shanghai, China

**Keywords:** cancer, immunotherapy, gut microbiota, dietary intervention, dietary fungi

## Abstract

Immunotherapies are recently emerged as a new strategy in treating various kinds of cancers which are insensitive to standard therapies, while the clinical application of immunotherapy is largely compromised by the low efficiency and serious side effects. Gut microbiota has been shown critical for the development of different cancer types, and the potential of gut microbiota manipulation through direct implantation or antibiotic-based depletion in regulating the overall efficacy of cancer immunotherapies has also been evaluated. However, the role of dietary supplementations, especially fungal products, in gut microbiota regulation and the enhancement of cancer immunotherapy remains elusive. In the present review, we comprehensively illustrated the limitations of current cancer immunotherapies, the biological functions as well as underlying mechanisms of gut microbiota manipulation in regulating cancer immunotherapies, and the benefits of dietary fungal supplementation in promoting cancer immunotherapies through gut microbiota modulation.

## 1 Introduction

The concept of cancer immunotherapy was first carried out by clinicians and immunologists centuries ago, but it had not been widely accepted as a practical option against cancer until recently (1). With the achievement of modern biomedical technologies, various types of immunotherapeutic strategies have been developed, which include the well-known “immune checkpoint blockade (ICB)” therapy (2), represented by the antibodies blocking the cytotoxic T-lymphocyte antigen-4 (CTLA-4) and the programmed cell death 1 (PD-1) and its ligand (PD-L1), as well as the “chimeric antigen receptor T cell (CAR-T)” therapy (3). Besides, many other immunotherapies were also proved to be efficient in treating certain types of cancers (4–7). However, the overall response of cancer patients to immunotherapies varies and serious symptoms were frequently observed, which dampened further utilization of this novel approach (8, 9). For instance, immune-related adverse events (irAEs) were frequently observed in patients receiving immune-checkpoint inhibitors (ICIs), characterized by excessive inflammation of multiple organs including skin, liver, lung, gastrointestinal tract, endocrine organs, etc. The incidence of irAEs varies among cohorts due to different type of ICIs and cancers (10), however, according to a retrospective study, 91.3% patients were affected by at least one type of irAE following ICI treatments (11). Cytokine release syndrome (CRS), a systemic inflammatory response, was closely associated with patients receiving CAR-T therapies. It was reported that the overall incidence of CRS can be as high as 90% in patients receiving CAR-T treatments (12). Therefore, a comprehensive understanding of the biology of cancer immunotherapy is required to improve its efficiency as well as to eliminate any potential side effects.

In recent years, the emerging role of gut microbiota in human health was emphasized with the development of multi-omic technologies (13). Previous studies have shown that the disruption of gut microbiota homeostasis contributes to the progression of various diseases (14–17). For cancer immunotherapies, the role of gut microbiota in regulating the efficiency as well as side effects of immunotherapy has also been revealed (18), as certain microbial species or related metabolites were shown closely correlated with the higher responsiveness of cancer patients. Nevertheless, the exact molecular mechanisms that how gut microbiota affect the host’s response to cancer immunotherapies are still under investigation.

Gut microbiota is composed of trillions of residing microbes, which are strongly affected by consumed nutrients (19); therefore, it is a potential way to manipulate gut microbiota through diet intervention to achieve a better outcome for cancer patients receiving immunotherapies (20). Numerous studies have been conducted on the nutritional value of plant or animal natural products as well as their modulation of gut microbiota and tumor immunotherapy (21, 22). Fungal products as a hot topic in recent years have attracted much attention due to their rich nutritional value and regulating functions to human body, of note, the regulatory role of fungal products on gut microbiota and cancer immunotherapies has been revealed (23). In this review, we will focus on the relationship between intake of natural products and gut microbiota modulation, as well as their biological role and underlying mechanism in cancer immunotherapies.

## 2 Advances and limitations of current cancer immunotherapies

### 2.1 Immune checkpoint blockade therapy

Compared with previous standards of care including chemotherapy, radiotherapy, and surgery, immunotherapy is a newly introduced approach, while showing significant improvement in the survival as well as the life quality of cancer patients (24). ICB is one of the most revolutionary technologies based on the theory of “immune surveillance” and the discovery of immune checkpoint molecules including CTLA-4 and PD-1, etc., on T cells (25, 26). Signals transduced by CTLA-4 following CD80/86 binding, and PD-1 following PD-L1 binding inhibit the “hyperactivation” of T cells and are important in preventing abnormal immune responses commonly seen in autoimmune diseases (27). However, these signals need to be abrogated to enhance the activity of T cells for the clearance of cancer cells (28). According to previous studies, patients treated with monoclonal antibodies against CTLA-4 or PD-1/PD-L1 resulted in dramatic antitumor responses through upregulation of immune activity (29–31). Mechanistic studies revealed that CTLA-4 or PD-1/PD-L1 blockade significantly enhanced T cell receptor (TCR) signals in tumor-specific T cells which leads to stronger tumor-killing activity (32), the infiltration as well as the survival rate of T cells in tumor microenvironment (TME) were also enhanced accordingly (33, 34). Currently, ICB has been approved for use in various types of cancers including melanoma, non-small cell lung cancer (NSCLC), renal cell carcinoma (RCC), squamous cell carcinoma of head and neck (SCCHN), bladder cancer, merkel cell carcinoma, hepatocellular carcinoma, Hodgkin lymphoma as either first-line or second-line treatment (35). Besides, many other agonistic and antagonistic immune checkpoint modulators which target co-stimulatory factors like 4-1BB, ICOS, GITR, OX-40, CD40, etc., are currently under investigation (36).

Along with the achievements in clinical practice, ICB therapy still has many limitations. One notable challenge is the generally low response across different types of cancers (37). Although the efficiency of anti-PD-1/PD-L1 has been clearly addressed in melanoma and NSCLC, the results from other types of cancers are less promising, and the response of individuals to ICB varies, which suggested the effects of other factors including genetics, environment, behavior, and even gut microbiota on the therapeutical efficiency of ICB (38). Another limitation of ICB is the associated side effects, named irAEs (39). IrAEs are excessive inflammatory responses induced by ICB therapies that multiple organs can be affected, even leading to death in some cases. It was reported that the overall irAEs incidence in ICB is around 70–90% (40). The most common symptoms of irAEs involving the skin, gastrointestinal tract, liver, endocrine organs, and lungs, while it varies among different types of cancers and therapies (41). For instance, colitis is the most common type of irAEs in the gastrointestinal tract, which occurred in 10-20% patients receiving ICI treatments (42). Cutaneous irAEs, including rash, pruritus and vitiligo, are also common-seen side effects in ICI therapies, of which approximately 50% patients were affected (43). The exact mechanisms for irAE development are still under investigation, while it was proposed that the over-activated T cell attacking normal tissue, uncontrolled secretion of cytokines, expansion of autoantibodies and even binding of ICI antibodies to normal tissues (the off-target effect) are responsible for the development of irAEs (44, 45).

Interestingly, it has been revealed that gut microbiota may affect the efficacy of ICB therapy as well as the incidence of associated irAEs (46, 47). Fecal material transplantation (FMT) has been shown effective in improving the overall response to PD-1 therapy in patients with melanoma or epithelial tumors, which indicates a substantial role of gut microbiota in modulating host immune response following PD-1 treatment (48, 49). On the other side, one study on melanoma patients receiving anti-CTLA-4 treatment showed that the enrichment of *Bacteroidetes* is strongly correlated with less frequency of colitis (50), which was supported by others (46, 47). Despite these findings, there is still an urgent need to improve the efficiency as well as eliminate the side effects of ICB, which relies on a deep understanding of the mechanism of the host response to ICB as well as an illustration of the interplay between host immune response, ICB as well as gut microbiota.

In recent years, bispecific antibodies treatment has been recognized as another promising approach in cancer immunotherapies (51). Notably, bispecific antibodies targeting both PD-1/PD-L1 and TGF-β named YM101 and M7824 were developed and achieved superior effects against cancers by overcoming the anti-PD1/PD-L1 drug resistance induced by TGF-β (52–54). Given the established interactions between gut microbiota and cancer immune responses, it would be also worthwhile to investigate the potential effects as well as underlying mechanisms of gut microbiota on the therapeutical efficiency of bispecific antibodies.

### 2.2 Chimeric antigen receptor therapy

As mentioned earlier, chimeric antigen receptor T cell (CAR-T) therapy is characterized by the genetic modification of T cells to strengthen their capability against cancer cells. Traditionally, T cell activation depends on the interaction between TCR and specific antigens (including tumor cell-associated antigens) presented by the Major Histocompatibility complex (MHC) on the cell surface, which is frequently down-regulated by tumor cells (55). To overcome this, a chimeric antigen receptor protein (which is composed of the ectodomain of cancer antigen-specific B cells and the intracellular domain of T cells) is designed and artificially expressed in normal T cells from patients to produce the CAR-T cells (56). Compared with the normal T cells, CAR-T cells exhibit much higher affinity as well as stronger killing activity against tumor cells both *in vitro* and *in vivo* (57, 58). CAR-T therapy was first developed for treating blood cancers including lymphoma and leukemia and exhibited promising results compared to conventional therapies (59). Currently, CAR-T therapies have been approved for treating various kinds of cancers including relapsed or refractory multiple myeloma, diffuse large B cell lymphoma (DLBCL), high-grade B-cell lymphoma, primary mediastinal large B-cell lymphoma, acute lymphoblastic leukemia (ALL), etc. (60). Additionally, the potential of CAR-T therapy in treating other types of cancers is also evaluated in both clinical and pre-clinical studies (61).

Nevertheless, there are also notable drawbacks of CAR-T therapy, and one of the most challenging issues is the development of tumor resistance to single antigen targeting CAR constructs. Although the administration of CAR-T cells initially yields high response rates, a significant proportion of patients experienced the loss of target antigen expression either partially or completely, which is known as antigen escape (62). It reported that 70–90% of ALL patients show durable responses to CD19 CAR-T therapy in the initial phase; however, it was followed by the downregulation or loss of CD19 antigen expression in 30–70% of the recurrent proportions (63). Consistently, downregulation of other targets including B-cell maturation antigen (BCMA) was also observed in CAR-T treated multiple myeloma patients (64). Another challenge of CAR-T therapy in clinic is the systemic cytokine release syndrome (CRS), which is characterized by hypotension, cardiac dysfunction, circulatory collapse, respiratory failure, renal failure, multiorgan system failure, etc., and may be life-threatening if not well-controlled (65). Pro-inflammatory IL-1 and IL-6 was identified as the key mediators of CRS in CAR-T therapies; therefore, IL-6/IL-6R blockade has been suggested as potential approaches to eliminate CRS. However, even with the use of tocilizumab, an FDA-approved IL-6R mAb in treating severe CRS, symptoms still persist and eventually lead to patient death (66). To date, an effective strategy against CAR-T therapy-induced CRS is still lacking. In addition, the efficacy of CAR-T therapy on solid tumors is compromised by low ability of tissue infiltration (67), which leads to less promising therapeutic outcomes (68). Localized injection instead of systemic administration was utilized to facilitate tumor infiltration of CAR-T cells, while it is only practical for single tumor lesions/oligometastatic disease (69).

The correlation between gut microbiota and the response/toxicity of CAR-T therapy was recently discovered. According to one cohort study, gut microbiome profile is strongly correlated with response and toxicity following anti-CD19 CAR T cell therapy in B cell malignancy patients, as revealed by distinct bacterial taxa and metabolic pathways in patients treated with/without antibiotics, as well as worse survival and increased neurotoxicity seen in patients exposed to antibiotics (piperacillin/tazobactam, meropenem and imipenem/cilastatin) (70). Nevertheless, there is still largely unknown regarding the role of gut microbiota on CAR-T therapy outcomes and the mechanistic insights are still lacking.

### 2.3 Other cancer immunotherapies

Beyond the mainstream cancer immunotherapies described above, there are also several other immunotherapies developed or under investigation. IL-2 is a typical example of cytokine therapies and was approved by FDA for treating metastatic renal cell carcinoma in 1992, while the significant toxicities including capillary leak syndrome and multiple organ dysfunction limit the use of IL-2 (71). T cell receptor-engineered T cell (TCR-T) therapy, another subtype of adoptive cell transfer (ACT) therapy just like CAR-T, is characterized by the genetic modification of T cells by implantation with a tumor antigen-specific TCR molecule. The advantage of TCR-T has been well documented in both pre-clinical and clinical studies (72). Beyond that, cancer vaccines and oncolytic virus therapies are also recognized as effective strategies for cancer (4, 5, 61). However, the role of gut microbiota in regulating the efficiency or toxicity of cancer immunotherapies still needs to be addressed.

## 3 Influence of gut microbiota on cancer immunotherapy

**Figure 1:**
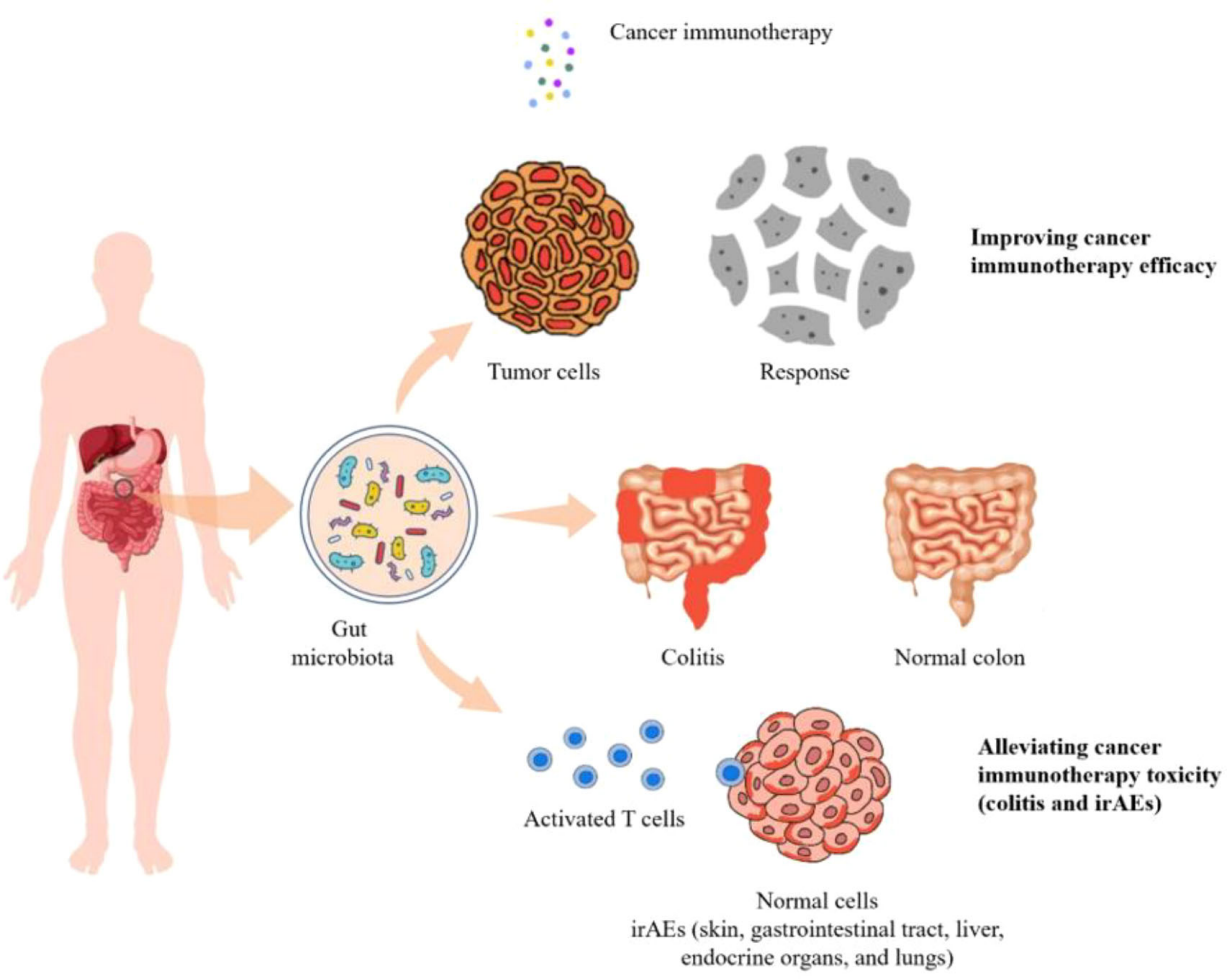
The diversity and composition of the gut microbiome can impact the efficacy as well as the toxicity of cancer immunotherapies including colitis and irAEs.

Gut microbiota is a complex community of microorganisms living in digestive tracts, which has the biggest quantities and greatest number of species compared to any other parts of the body (73). It is well recognized that microbes in human gut play fundamental roles in the well-being of the host. Interactions within constituents of the microbiota (bacteria, viruses, and eukaryotes) as well their relationship with the host immune system influence the development of disease in many ways. For example, it protects the host from pathogens by colonizing mucosal surfaces and secreting various antimicrobial substances, which help enhance the immune response (74). In addition, gut microbiota plays a vital role in digestion and metabolism, controlling epithelial cell proliferation and differentiation, regulating insulin resistance, and brain-gut communication (75). With respect to cancer immunotherapies, the composition, biological activity, and metabolic products derived from gut microbiota were shown to have substantial impacts on efficiency as well as side effects of treatments (**Figure 1**).

### 3.1 Gut microbiota affects the efficiency of cancer immunotherapies

The relationship of gut microbiota and anti-PD-1 efficacy in melanoma has been revealed by previous studies. Sivan et al. examined the subcutaneous growth of B16.SIY melanoma in genetically similar C57BL/6 mice raised in two facilities (76). They found that the tumor growth was more aggressive in one group, which was associated with significantly lower intra-tumoral CD8^+^ T cell accumulation and was affected by gut microbiota composition. In line with this, Gopalakrishnan et al. examined the gut microbiota of melanoma patients undergoing anti-PD-1 therapy and observed a significant difference in the diversity and composition of the gut microbiota between responders and non-responders (48). Additionally, a retrospective cohort study found that exposure to antibiotics in four weeks before CAR-T therapy might reduce patients survival and increase the incidence of neurotoxicity, which underscored the association between gut microbiota and the efficiency of CAR-T therapy (72). The effects of gut microbiota on CAR-T therapy were also proposed and further supported by the observation of close correlation between gut microbial composition and the response to CAR-T therapy (70, 77–79).

The involvement of specific gut microbial species in cancer immunotherapies has also been accessed. Matson et al. examined the stool samples collected from patients with metastatic melanoma before anti-PD-1 immunotherapy and found that *Bifidobacterium longum*, *Collinsella aerofaciens*, and *Enterococcus faecium* were more abundant in responders, indicating the antitumor effects of *Bifidobacterium* species in the context of PD-1 immunotherapy (80). Similarly, previous study showed that the antitumor effects of CTLA-4 blockade depend on distinct *Bacteroides* species, as T cell responses specific for *B. thetaiotaomicron* or *B. fragilis* were associated with the efficacy of CTLA-4 blockade (81).

To determine the biological role of gut microbiota in regulating patients’ response to ICB therapy, Davar et al., evaluated the therapeutic efficiency of combined treatment of FMT (from PD-1 responders) and anti-PD-1 administration on patients with PD-1 refractory melanoma (82). It showed that the responders exhibited an increased abundance of taxa that were previously shown to be associated with response to anti–PD-1, increased CD8^+^ T cell activation, and decreased frequency of IL-8 expressing myeloid cells. Additionally, responders had distinct proteomic and metabolomic signatures, and trans-kingdom network analysis revealed the dominant role of gut microbiota in regulating these changes. These results confirmed the effect of gut microbiota in improving anti-PD-1 efficiency against melanoma. In addition to this, gut microbiota had also been found critical in enhancing the efficacy of PD-1 therapy against other cancer types (83).

The biological role of certain gut microbial species in regulating the efficiency of cancer immunotherapies has been addressed by previous studies. It was revealed that *Bifidobacterium* species were beneficial in promoting antitumor immune responses during anti-PD-1 treatment (76). The cause-effect relationship between gut colonization of *B. fragilis* and improved outcome of CTLA-4 blockade therapy has been well demonstrated by fecal material transplantation, and *B. fragilis* implantation approaches (81). In a similar study, Routy et al., uncovered the role of *Akkermansia muciniphila* in regulating the response of melanoma patients to anti-PD-1 treatment (49).Oral supplementation with *A. muciniphila* after fecal material transplantation (FMT) with feces from non-responders significantly restored the efficacy of PD-1 blockade (49). Altogether, these findings highlight the importance of gut microbiota manipulation in improving the efficiency of cancer immunotherapies.

### 3.2 Gut microbiota affect the toxicities of cancer immunotherapies

Another question regarding gut microbiota in cancer immunotherapies is how the alteration of gut microbes, either in composition or in biological activities, affect the risk of ICB associated toxicities. It showed that the composition of intestinal microbiota can predict whether a patient will develop colitis following treatment with ipilimumab, a monoclonal antibody that blocks CTLA-4 (50). 16S rRNA gene sequencing/16S rDNA sequencing results showed that both the colitis and colitis-free groups had similar microbial compositions before the onset of colitis by ipilimumab administration, however, patients who remained colitis-free following treatment showed a higher proportion of *Bacteroidetes* phylum. Specifically, *Bacteroidaceae*, *Rikenellaceae*, and *Barnesiellaceae* were more abundant in the feces of colitis resistant patients. Metagenomic sequencing analysis further revealed that 4 microbial modules associated with polyamine transport and B vitamin biosynthesis were more abundant in the microbiota of patients who remained colitis-free. In line with this, gut microbiota can also be used for the prediction of side effect risks in anti-PD-1/PD-L1 therapies. According to an observational study, patients with severe irAEs showed higher abundance of *Streptococcus*, *Paecalibacterium*, and *Stenotrophomonas*, while patients with mild irAEs were enriched in *Faecalibacterium* and *Lachnospiraceae* (84). Similarly, another clinical study revealed the distinct compositional differences in gut microbiota between patients who experienced clinically significant or non-significant irAEs (85). In a more comprehensive study led by Dr. Jennifer Wargo, blood, tumor, and gut microbiome of 77 patients with advanced melanoma treated with combined immune checkpoint blockade (CICB) targeting PD-1 and CTLA-4 were profiled, and a significantly higher abundance of *Bacteroides intestinalis* was found in patients with toxicity characterized by colitis and upregulation of mucosal IL-1β (47). In addition, as mentioned above, exposure to antibiotics was correlated with a high incidence of neurotoxicity in B cell lymphoma and leukemia patients receiving CD19 CAR-T therapy (70). This indirectly confirmed that the gut microbiota could alleviate the related side effects of CAR-T therapy.

As another common side effect of ICB, colitis is commonly treated with immunosuppressive drugs, including corticosteroids and/or agents targeting tumor-necrosis factor-α (TNF-α), all of such have obvious side effects (86). Clinical evidence showing that colitis and inflammatory bowel disease (IBD) can be successfully treated by gut microbiota manipulation aroused the interest to develop new strategies against ICB-induced colitis (87, 88). It revealed that FMT treatment significantly reduced the incidence and severity of colitis in patients receiving ICB therapies, with a relative increase in the proportion of regulatory T-cells within the colonic mucosa area, although the sample number is too limit (n=2) to draw a firm conclusion (46).

Overall, the biological roles of gut microbiota in regulating efficacy as well as toxicity of cancer immunotherapies have been well documented, which is indicative the future development of new strategies in boosting cancer immunotherapy.

## 4 Dietary fungi in cancer immunotherapies

### 4.1 Overview of dietary products, gut microbiota, and cancer immunotherapy

With a deeper understanding of gut microbiota, potential ways to optimize gut microbiota in patients and healthy people have also been evaluated recently. Fecal microbiota transplantation (FMT) and single bacteria transplantation (probiotic administration) have achieved promising results in improving the wellness of patients, however, it may be detrimental for patients exposed to the allogenic strains for FMT and make people vulnerable to chronic diseases such as autoimmune disease (AID) (89, 90). Instead, diet intervention or prebiotic supplementation may be more suitable for the general population as it is considered less harmful and easier to be accepted (91). Meanwhile, the regulatory mechanism of diet intervention on gut microbiota is necessary to be elucidated as a pre-requirement. Researchers have been working in this field for decades and many dietary supplements were identified to have “microbiota-modulating” activity. In general, animal-based diets resulted in higher levels of amino acid fermentation products and lower levels of carbohydrate fermentation products, while the levels of amino acid fermentation products were positively correlated with the amounts of putrefactive, bile-tolerant microbes like *Bacteroides* and *Clostridia*, saccharolytic microbes were increased as well. In contrast, beneficial bacteria, like *Bifidobacteria* and *Eubacteria* were negatively correlated with the consumption of animal products (92). In line with this, high-fat and animal-based diets can promote the growth of *Bilophila wadsworthia*—a bacteria producing hydrogen sulfide (H_2_S), which is presumably responsible for GI inflammation (93). Nevertheless, high consumption of polyunsaturated fats fosters *Ruminococcus* growth inside the gut (94). The role of fiber in plant-based diets has been revealed by several studies. For instance, diets rich in carbohydrates and fiber increase the variety and richness of gut microbiota, characterized by the increase of *Bacteroidetes* and decrease of *Firmicutes*/*Bacteroidetes* ratio (95). High fiber intake also boosted the outgrowth of *Firmicutes* and *Proteobacteria*, which were usually low in subjects consuming high-fat diets (96). In contrast to that, the high intake of simple sugar instead of fiber resulted in a substantial outgrowth of *Bacteroides* (97). Vegetarian diets do not contain any meat or fish while rich in carbohydrates and fiber. Consumption of vegetarian diets led to increased production of short chain fatty acids (SCFA), which is beneficial in preventing GI inflammation and maintaining the homeostasis of microbial flora in the gut (98). Moreover, it was found that protein consumption increases the diversity of gut microbiota; however, the effects vary depending on the source of proteins. Notably, whey and pea protein consumption increased the levels of *Bifidobacterium* and *Lactobacillus*, while limiting the growth of *Bacteroides fragilis* and *Clostridium perfringens*. In addition, pea protein increased the level of SCFA in GI levels. Meanwhile, an animal-protein-based diet stimulates bile-tolerant anaerobes (90).

Along with the assessment of the role of animal or plant-based diets on gut microbiota, modulating activity of phytochemicals as well as prebiotics on gut microbiota was also evaluated. Phytochemicals, including polyphenols, carotenoids, phytosterols/phytostanols, lignans, alkaloids, have been shown to have positive effects on the modulation of gut microbiota (99). Supplementation of carotenoids such as astaxanthin or retinoic acid help maintains intestinal immune homeostasis by inducing IgA production (100). One study showed that bilberry anthocyanin consumption promoted the efficiency of ICB by modulating the composition of gut microbiota (21). They found that the antitumor efficiency of anti-PD-L1 antibody was enhanced by oral administration of bilberry anthocyanin extracts in a mouse tumor model, which was accompanied by the enrichment of *Clostridia* and *Lactobacillus johnsonii* in feces. Prebiotics, including fructooligosaccharides, galactooligosaccharides, soybean oligosaccharides, inulin, etc., exert benefits in the regulation of gut microbiota by increasing the population of commensals like *Lactobacillus* and *Bifidobacterium* (100). One study reported that orally administered inulin gel could enhance the antitumor efficacy of α-PD-1 therapy *via* modulating commensal microorganisms in situ, as well as lead to a potent long-lived antitumor effect *via* eliciting memory CD8+ T-cell responses (101). Overall, despite interesting findings on natural products from plants and animals, few studies have investigated natural products from dietary fungus, which may also have potential effects on the regulation of composition as well as biological activity of gut microbes.

### 4.2 Impact of dietary fungi on gut microbiota

Compared with extensive research on the effect of plants or animal-derived diets on gut microbiota regulation, the role of dietary fungus remained unexplored until recently. *Lentinula edodes* (shiitake) is an edible mushroom enriched with different types of polysaccharides (102), it showed that administration of heteropolysaccharides from *Lentinula* significantly altered the diversity of microbiota in the small intestine, cecum, colon, and the distal colon (feces) in mice (103). Specifically, the decrease of *Bacteroidetes* was associated with the increase of *Proteobacteria*. Of note, *Chloroflexi*, *Gemmatimonadetes*, *Nitrospirae*, and *Planctomycetes* are exclusively present in treated mice. One study reported that *Lentinula edodes* by-products (LESDF-3) could promote the production of *Bacteroides*, indicating the importance of LESDF on the regulation of gut microbiota (104). In line with this, several studies also demonstrated the biological functions of polysaccharides from *Cordyceps Sinensis*, *Auricularia auricular-judae*, *Ganoderma lucidum*, *Grifola frondose, Pleurotus ostreatus*, *Hericium erinaceus*, and wild morels in reshaping gut microbiota and immune regulation (105–111). *Ganoderma lucidum* has long been considered valuable in medication and diet supplementation. According to a recent study, spore oil from *Ganoderma lucidum* has strong immunoenhancing activity, which was correlated with the elevated abundance of several bacterial genera (*Lactobacillus*, *Turicibacter*, and *Romboutsia*) and species (*Lactobacillus*_*intestinalis* and *Lactobacillus_reuteri*), as well as reduced level of *Staphylococcus* and *Helicobacter* (112). Those alterations in gut microbiota further resulted in the secretion of a range of key metabolites such as dopamine, prolyl-glutamine, pentahomomethionine, leucyl-glutamine, L-threonine, stearoylcarnitine, dolichyl β-D-glucosyl phosphate to enhance phagocytosis of macrophages and NK cell cytotoxicity. Inulin, a type of natural polysaccharide that exists in various kinds of natural products has been recognized as a powerful probiotic (113), interestingly, β-glucan from dietary fungus also exhibited comparative effects with inulin (114). Specifically, β-glucan could modulate the structure and composition of gut microbiota by inhibiting the proliferation of harmful gut microbiota while upregulating the abundance of beneficial *Bacteroidetes*. Furthermore, both β-glucan and inulin could selectively promote the growth of *Bifidobacterium*. D-glucan from mushrooms also showed similar effects on gut microbiota modulation and thus it can be considered a new type of probiotic. A list of dietary fungi and their roles in modulating gut microbiota is summarized in **Table 1**.

**Table 1:** List of dietary fungi, the bioactive components, and their role in modulating gut microbiota.

Name	Bioactive components	Biological effect on gut microbiota	Reference
*Lentinula edodes*	LESDF	LESDF-2: upregulating the abundance of *Faecalibacterium* and *Bifidobacterium*; LESDF-3: *Bacteroides*, *Parasutterella*, *Parabacteroides* and *Lachnospira*	(104)
*Cordyceps Sinensis*	Polysaccharides	Downregulating the abundance of *Bacteroidetes*. Upregulating the abundance of *Proteobacteria*, *Actinobacteria* and *Acidobacteria*	(105)
*Auricularia auricular-judae*	Polysaccharides	Downregulating the abundance of *Ruminococcus*, *Deferribacteres* and *Actinobacteria* compared to control (IBD model)	(106)
*Ganoderma lucidum*	Polysaccharides	Upregulating the abundance of *Blautia*, *Dehalobacterium*, *Parabacteroides*, and *Bacteroides*, while downregulating the levels of *Aerococcus*, *Ruminococcus*, *Corynebacterium* and *Proteus*	(107)
*Grifola frondose*	Pseudobaptigenin and cyanidin 3-o-xylosylrutinoside	Upregulating the abundance of *Paraprevotella*, *Porphyromonadaceae*, *Anaerotruncus*, *Barnesiella*, *Parasutterella*, and Desulfovibrionaceae.	(108)
*Pleurotus ostreatus*	Polysaccharides	Upregulating the abundance of α-proteobacteria and γ-proteobacteria	(109)
*Hericium erinaceus*	Polysaccharides, peptides, crude fat, etc.	Upregulating the abundance of *Bifidobacterium*, *Bacteroides* and SCFAs-producing bacteria such as *Roseburia* and *Faecalibacterium*, etc.	(110)
*Wild morels*	Polysaccharides	Upregulating the abundance of *Bacteroidetes*, *Lachnospiraceae* and the total levels of the SCFA-producing bacteria *Lachnospiraceae*, *Ruminococcaceae*, *Erysipelotrichaceae*	(111)
*Ganoderma lucidum*	Triterpenes and fatty acids	Downregulating the abundance of *Firmicutes* while upregulating the abundance of *Bacteroidete.*	(112)

### 4.3 Impact of dietary fungi on cancer immunotherapy

Despite the well-known effects of plant-based or animal-based diets on cancer immunotherapy, the relationship between dietary fungus and the efficiency of cancer immunotherapy remains elusive. Previous studies have investigated the role of key components from dietary fungus in cancer immune regulation. The main component of the fungal cell wall is β-Glucan, which has been reported to function as potent immunomodulators to enhance antitumor immune responses by regulating differentiation and function of monocytic myeloid-derived suppressor cells (MDSCs) (115). Consistently, polysaccharides from *Agaricus blazei Murill* stimulated MDSC differentiation from M2 to M1 type, which mediates inhibition of tumor immune evasion *via* the Toll-like receptor 2 (TLR2) (116). It was later revealed that natural killer (NK) cells, macrophages, and dendritic cells are responsible in mediating fungal products originated antitumor immune response. According to one study, *Agaricus bisporus* polysaccharides MH751906 exerted immunotherapeutic effect on colon cancer by activating gut-residing NK cells, and these activated NK cells played a stable role in killing human colon cancer cells (117). In line with this, another study reported that *Boletus edulis* RNA could also stimulate NK cells against myelogenous leukemia (118). In addition, one study indicated that polysaccharides from *Luchnum* boosted antitumor immune responses by resetting tumor-associated macrophages (TAMs) from the “pro-tumor” M2 to the “antitumor” M1 phenotype (119). Another study uncovered the immunomodulatory activity of polysaccharide−protein complex from *Polyporus rhinocerus*, exerting antitumor effects by activating macrophage-mediated host immune response (120). *Ganoderma lucidum* polysaccharides could partially or completely antagonize the suppression of B16F10 melanoma cells on the viability of peritoneal macrophages, suggesting its potential role in cancer immunotherapy (121). Following study found that the antitumor effect of *Ganoderma lucidum* was from the stimulation of dendritic cell maturation and initiation of the adaptive immune response towards T helper 1 (Th1) polarization *in vivo* (122). Both studies indicated the immunomodulatory mechanism mediated by *Ganoderma lucidum* in the antitumor process. Additionally, polysaccharopeptide (PSP) extracted from *Coriolus versicolor* showed immunotherapeutic effects against tumors *via* strengthening the phagocytosis of macrophages, increasing the expression of cytokines and chemokines, as well as stimulating the infiltration of both dendritic cells and T-cells into tumors (123).

The exact role of dietary fungi in cancer immunotherapy is still largely unknown except for a few studies reported the relevant effects. One study revealed *Cordyceps militaris* polysaccharide converted immunosuppressive macrophages into M1 phenotype and activated T lymphocytes by inhibiting the PD-1/PD-L1 axis between TAMs and T lymphocytes, which may improve the effectiveness of anti-PD-1/PD-L1 immunotherapy (124). Another study showed that *Ginseng* polysaccharides altered the gut microbiota and kynurenine/tryptophan ratio, potentiating the antitumor effect of PD-1/PD-L1 immunotherapy (125), which elucidated the axis of action of fungal polysaccharides through the gut microbiota to strengthen the antitumor effects of ICB. In addition to enhancing the therapeutic effect of ICB, taking fungal products has also been reported to improve the efficacy of cancer vaccines. Oral ingestion of *Lentinula edodes* mycelia extracts could enhance the antitumor activity of peptide vaccine, which indicated that *Lentinus edodes* extracts play a vital role in cancer immunotherapy (126). Collectively, it is of particular interest to comprehensively understand the role as well as the molecular mechanism of dietary fungus in cancer immunotherapy, and the involvement of gut microbiota in this process also needs to be further addressed. A list of dietary fungi and their roles in modulating host immune response against cancers is summarized in **Table 2**.

**Table 2:** Biological roles and mechanisms of dietary fungi in treating various types of cancers.

Name	Cancer type	Biological effects on host immune response	Reference
*Agaricus blazei Murill*	Cervical cancer	*Agaricus blazei Murill* Polysaccharide selectively block the Toll-like receptor 2 (TLR2) signal to stimulate myeloid-derived suppressor cell differentiation from M2 to M1 type.	(116)
*Agaricus bisporus*	Colon cancer	*Agaricus bisporus* polysaccharides activate gut resident natural killer cells.	(117)
*Boletus edulis*	Myelogenous leukemia	*Boletus edulis* RNA fraction enhances NK cell activity against Myelogenous Leukemia Cells.	(118)
*Luchnum*	Sarcoma	*Lachnum* polysaccharide resets TAMs from pro-tumor M2 to anti-tumor M1 phenotype, resulting in the accumulation of anti-tumor immune cells while decreasing the infiltration of immunosuppressive cells such as myeloid-derived suppressor cells (MDSCs) and Treg cells.	(119)
*Ganoderma lucidum*	Melanoma	*Ganoderma lucidum* suppresses the viability, phagocytic activity, NO production, TNF-α production and activity in peritoneal macrophages suppressed by B16F10.	(121)
*Ganoderma lucidum*	Colon adenocarcinoma, melanoma, and sarcoma	Purified polysaccharide fraction from *Ganoderma lucidum* exerted antitumor effects by stimulating dendritic cell mature and initiating adaptive immune response towards T helper 1 polarization *in vivo*	(122)
*Coriolus versicolor*	Not specified	*Coriolus versicolor* poly-saccharopeptide promotes the phagocytosis of macrophages, increasing the expression of cytokines and chemokines, and stimulating the infiltration of both dendritic cells and T-cells into tumors.	(123)
*Cordyceps militaris*	Breast cancer and lung cancer	*Cordyceps militaris* polysaccharides reset TAMs from a tumour-promoting M2 phenotype to a tumour-killing M1 phenotype by inhibiting the PD-L1/PD-1 axis between TAMs and T lymphocytes to reverse the functional inhibition of T lymphocytes.	(124)
*Ginseng*	Lung cancer	*Ginseng* polysaccharides enhance the efficacy of αPD-1 mAb by regulating gut microbiota and decreasing L-kynurenine, as well as the ratio of Kyn/Trp.	(125)
*Lentinula edodes*	Melanoma	*Lentinula edodes* mycelia extract enhances peptide vaccine-induced anti-tumor activity by inhibiting the increase of the percentage of Tregs in tumor-bearing hosts.	(126)

### 4.4 Clinical studies for dietary fungi on cancer immunotherapy

To date, a few clinical studies have been conducted to evaluate the antitumor activities of fungal products and the underlying mechanisms. It showed that *Grifola frondose* could inhibit lung or breast cancer metastasis and decrease the size of tumors (127), which was achieved by increasing NK cell activity and promoting Th1 response, while with the reduced Th2 activity. Another study showed that *Ganoderma lucidum* polysaccharide has antitumor effects on various types of advanced-stage cancer, which was achieved by stimulating host immune response, including the increased secretion of IL-2, IL-6, IFN-γ, and enhanced NK cell activity, whereas the concentration of IL-1β and TNF-α was reduced compared with baseline (128). According to a study conducted by Zhao et al., 48 breast cancer patients were treated with *Ganoderma lucidum* spore powder, and it was shown that the concentrations of TNF-α and IL-6 in the serum of patients after treatment decreased significantly, which was accompanied by reduced tumor burden (129). In addition, *Pleurotus cornucopiae* (Oyster mushroom, Tamogitake) was also found to have antitumor effects through host immune regulation. Tanaka et al. conducted a clinical trial to investigate the antitumor activity of *P. cornucopiae*, and it was found that the levels of serum IFN-γ and IL-12 increased along with NK cell activity, suggesting the involvement of Th1 immune response in *P. cornucopiae* directed antitumor activity (130). The antitumor effects on adenocarcinoma as well as the immunomodulation activity of active hexose correlated compound (AHCC, obtained from *Lentinus edodes* of Basidiomycete mush) was also evaluated clinically, it showed that AHCC treatment led to the increase of neutrophils, the ratio of CD3^+^/CD4^+^, CD4^+^/CD8^+^, CD3^+^/CD16^+^/CD56^+^ NK cells were also increased accordingly, while the number of lymphocytes and monocytes were reduced (131). In addition, a phase I trial demonstrated the antitumor effect of *Agaricus bisporus* on prostate cancer by modulating IL-15 level and MDSC activity (132). Recently, several clinical trials are underway to evaluate the antitumor effect of additional fungal extracts and to explore the underlying immunomodulatory mechanisms, while the results have not been reported yet (133). Overall, the antitumor effects of fungal products as well as the molecular mechanisms are well documented, however, most conclusions are drawn only based on small samples. Furthermore, though clinical studies have validated that fungal products can enhance the antitumor effects of chemotherapy and radiotherapy (133), clinical evidence regarding the efficacy of fungal products in cancer immunotherapies is still lacking. Therefore, additional trials are required to investigate the clinical effect of fungal products on cancer immunotherapies. A list of current clinical studies investigating the effects of dietary fungi in different cancers is summarized in **Table 3**.

**Table 3:** Clinical evidence of dietary fungi in treating different types of cancers.

Name	Cancer Type	Type of study	Main results	Reference
*Maitake (Grifola frondosa)*	Lung and breast cancer, stage II– IV	Interventional clinical trial, n=10	Maitake D-Fraction could inhibit lung or breast cancer metastasis and decrease the size of tumors, and NK cell activity was significantly elevated.	(127)
*Ganoderma lucidum*	Advanced-stage cancers arising from various tissues	Interventional clinical trial, n=34	Enhanced host immune responses (a significant increase in IL-2, IL-6, IFN-γ,CD56+ cells and NK activity compared with baseline) in patients with advanced-stage cancer.	(128)
*Ganoderma lucidum*	Breast cancer patients with cancer-related fatigue undergoing endocrine therapy	RCT, n=48	Significant decrease in TNF-α and IL-6 in serum, beneficial effects on cancer-related fatigue and quality of life compared to placebo.	(129)
*Lentinula edodes*	Adenocarcinoma (pancreatic, lung, colorectal)	Interventional clinical trial, n=7	A consistent increase in neutrophils, the ratio of CD3+/CD4+, CD4+/CD8+, CD3+/CD16+/CD56+ NK cells, while decrease in lymphocytes and monocytes	(131)
*Agaricus bisporus*	Prostate cancer	Single-arm, unblended, single-facilit, phase I trial (n=36)	Promising effect against prostate cancer by modulating IL-15 level and MDSC activity.	(132)

## 5 Conclusion

With the advanced achievement of high-throughput multi-omic technologies including microbial amplicon, meta-genomic, meta-transcriptomic, metabolomic and interatomic analysis in recent years, the biological relevance of gut microbiota on human health has been well recognized. Preclinical studies demonstrated the diverse functions of gut microbe in regulating host immune homeostasis, which is beneficial to protect against many diseases, and in particular, the improvement of cancer immunotherapies. Clinical studies again demonstrated gut microbiota modulating strategies in promoting the outcome of patients. Despite the discovery of various beneficial or harmful microbes/metabolites, however, the underlying mechanism remains unclear, which dampens further development of precise manipulation of gut microbiota. FMT has been proven to be effective in preclinical and clinical studies, while concerns regarding the safety of transferring fecal materials still exist. In 2019, it was reported that 2 patients receiving FMT treatment developed invasive infections caused by multidrug-resistant organisms (MDRO) and one of the patients eventually died (134). FDA of United Stated warned about potential risk of serious infections caused by MDRO related to the investigational use of FMT (135). Therefore, it is necessary to keep alert to FMT therapy–induced adverse events in further clinical investigation. Diet interventions, on the side, are much less concerning as it has been long accepted as common sense, but the effectiveness in gut microbiota modulation has not been well addressed. To solve this problem, studies have tried to identify the “key gradients” of daily diet or supplements, and polysaccharides, or “diet fiber” was eventually demonstrated to be essential for promoting the growth of commensals and maintaining a healthy gut environment; saturated acid from animal products, on the side, were shown negatively impact the gut microbiota characterized by the outgrowth of harmful microbes and production of pro-inflammatory factors. Regardless, it remains elusive for the effects of many other components, the underlying mechanism has yet to be clarified as well.

Immunotherapies have been demonstrated successful in treating various types of cancers and the improvement of patients’ well beings. Meanwhile, the effectiveness varies across individuals along with serious side effects such as neurotoxicity, cytokine release syndrome, colitis, etc. Since gut microbiota was shown closely related to clinical features of cancer patients upon treatment, it is applicable to predict the outcome of any individuals by accessing their gut microbiota. In fact, previous studies have demonstrated the efficiency of such strategies. Beyond that, gut microbiota has also been shown important in regulating the immune response of the host, which highlights its potential role in cancer immunotherapies. To achieve this, many studies have revealed the biological functions of the specific gut microbe and/or metabolites in boosting cancer immunotherapies both pre-clinically and clinically by FMT or single strain administration. Nevertheless, how diet intervention affects the behavior of gut microbiota and how is it related to cancer immunotherapy remains unclear. These questions need to be addressed by molecular identification of effective factors from diet and the validation of their functions by pre-clinical and clinical interventions.

Fungi have long been used in diet and medications, while their effects on gut microbiota had not been noticed until recently (102). Like plant or animal products, edible fungi contain various types of nutrients which can be recognized by gut microbes. As mentioned, the roles of dietary fungi on gut microbiota were discovered and the effective components were also pinpointed in recent studies. However, systemic screenings of fungi-derived components on gut microbiota are still lacking, which is the pre-requirement for a comprehensive understanding of the interplays between dietary fungi and gut microbes, and more studies are needed to identify the underlying mechanism. Ultimately, investigations of dietary fungi and gut microbiota will pave the way to developing therapies against cancer in combination with current immunotherapeutic approaches.

## Author contributions

YW, DS, HW, and XL made substantial contributions to the design, data collection, and writing of this manuscript. RW and TL assisted in data evaluation and helpful discussion. HW and XL supervised the project. All authors contributed to the article and approved the submitted version.
